# The Association Between Lunar Phases and Calving Frequency in Montbéliarde Dairy Cows in the Franche-Comté Region, France

**DOI:** 10.3390/ani16101431

**Published:** 2026-05-08

**Authors:** Juline Stoffel, Thomas Mercky, Ana Paiva, Anna Brasileiro

**Affiliations:** 1 CIVG—Vasco da Gama Research Center, EUVG—Vasco da Gama University School, 3020-210 Coimbra, Portugalana.paiva@euvg.pt (A.P.); 2 Clinique Veterinárie du Vernois, 7 Chem. des Alamans, 39270 Orgelet, France

**Keywords:** calvings, dairy cattle, Montbéliard, lunar cycle, lunar association

## Abstract

The Moon is at the centre of many popular beliefs, including the notion that the number of parturitions increases during Full Moon days, a belief widely held among breeders to anticipate calving periods. However, this association has been rarely explored in dairy cattle farming. This retrospective study was conducted to evaluate the association between the lunar cycles and calving distribution, with particular focus on a potential increase during full-moon nights. Data from 383,926 calvings of Montbéliard breed, recorded between March 2022 and January 2025, mostly in Franche-Comté (98.2%), France, were analyzed using a Generalized Linear Model (GLM). Results revealed a significant association between the lunar cycle and calving distribution. Model-adjusted means were significantly higher than the overall mean during the New Moon and the Last Quarter phases (+2.6%, *p* < 0.05), and significantly lower during the Full Moon phase (−3.1%, *p* < 0.01). At the synodic day level, days 21 and 29 presented the highest model-adjusted means (+23.2% and +23.4% above the overall mean, respectively; *p* < 0.001), while days 4 and 15 presented the lowest (−35.2% and −31.1%, respectively; *p* < 0.001). Across all seasons, a consistent trend toward increased calving frequency was observed during the New Moon phase, reaching statistical significance only in Spring (+5.7%, *p* < 0.05). These findings may offer potential implications for veterinarians and breeders, particularly when considering approaches to ensure adequate colostrum intake, which may contribute to improving management of parturition periods.

## Simple Summary

The belief that more animals are born during the Full Moon nights is widespread, but it has rarely been investigated in dairy cattle. In this study, nearly 384,000 calvings of Montbéliard cows in eastern France were analyzed to determine whether the lunar cycle is associated with calving patterns. The results showed that the highest number of calvings occurred during the New Moon, while the lowest numbers were recorded during the Full Moon. An increase in parturitions was observed between the New Moon and the Waxing Crescent, followed by a decrease toward the First Quarter. These findings indicate that the lunar cycle has a measurable effect on calving distribution, although not in the way traditionally assumed in this region. Recognizing these patterns may help veterinarians and farmers anticipate variations in calving distribution and consider them when planning neonatal care.

## 1. Introduction

Since ancient times, the relationship between the Moon and the number of parturitions or the onset of labor has been a source of much questioning, both in human and veterinary medicine.

In rural areas, many cattle breeders use the phases of the Moon to determine the final period of their animals’ gestation, believing that the Full Moon increases the number of parturitions [1,2]. Unlike in human medicine, few studies have been conducted in cattle breeding to verify this phenomenon. Among the existing studies, some have shown a relationship between the Moon and calving frequency, especially in populations of dairy cows [1] and dual-purpose crossbred cows [3,4,5], while others tend to refute it, as is the case with beef cow populations [6].

In cows, physiological calving results from a coordinated series of events culminating in birth, with the timing primarily determined by the fetus. Parturition commences when the near-term fetus signals its readiness: as fetal maturation completes, the fetal hypothalamic–pituitary–adrenal axis triggers a surge of fetal cortisol, which in turn induces a cascade of maternal endocrine changes [7,8]. This fetal cortisol surge causes a shift from progesterone dominance to an estrogen- and prostaglandin-dominated hormonal environment [9], effectively withdrawing the progesterone block on the uterus. The increase in estrogens and PGF2α, along with a late-gestational rise in relaxin, leads to accelerated cervical ripening and activation of myometrial contractility [10,11,12]. In the final weeks of gestation, the cow’s cervix gradually softens under rising estrogen (and relaxin) influence, with increasing hydration and collagen disorganization in cervical tissues as term approaches [7].

Clinically, the first stage is cervical dilation, which allows fetal alignment. The increasing pressure of the calf and fetal membranes against the cervix stimulates stretch receptors, provoking oxytocin release from the dam’s posterior pituitary and initiating the Ferguson reflex [12]. Oxytocin, together with elevated estrogen and prostaglandin levels, intensifies uterine contractions in a positive feedback loop. As contractions become organized and forceful, the cow enters the second stage, the expulsive phase. During this stage, strong coordinated myometrial contractions, along with the cow’s voluntary abdominal straining, propel the calf through the birth canal [7]. The expulsive phase results from the interaction of mechanical forces with hormonal control exerted by estrogens/PGF2α and relaxin [11,12].

The final stage of labor is characterized by the expulsion of the fetal membranes, where the fetal cotyledons separate from the maternal caruncle crypts [12,13,14]. In summary, the timing of birth in cows is a finely tuned interplay between fetal signals and maternal physiological preparedness, ensuring that delivery occurs when the calf is mature and the dam’s birth canal is optimally primed. Studies have shown that melatonin can have a positive effect on reproductive cycles [15,16] and that it plays a role in gestation duration and, therefore, in calving date [17].

The signs before labor are diverse, including physical changes as well as behavioral ones, such as a cow becoming more anxious and tending to isolate itself [13]. Montbéliarde breed animals are known for their calm temperament, being docile, and thus do not need to isolate themselves to calve [18].

This study is based on the analysis of secondary data from bovine calvings in the Franche-Comté region, France. This region is considered rural, as 95% of its area consists of rural properties [19]. Milk production represents a significant portion of the region’s economy, being used to produce cheese. Given the socioeconomic importance of dairy activity in the region, special attention must be given to herd health management, including possible assistance during primiparous calvings due to the higher frequency of dystocia [20,21,22], and care for newborns, such as colostrum feeding. Proper management in the first hours of the animal’s life has a positive impact on its health, influencing its productive capacity throughout its life [23,24,25].

To study the possible association between the Moon and calvings, some knowledge about the Earth’s only natural satellite is necessary [26]. The Moon orbits the Earth in an elliptical translational movement [27], performing several revolutions, the most studied being the synodic revolution, which lasts 29.53 days [28]. This corresponds to the time the Moon takes to orbit the Earth, compensating for the Earth’s movement around the Sun [29], representing the interval between two consecutive New Moons [30].

The lunar cycle consists of several distinct phases corresponding to the Moon’s apparent illumination, which is determined by the relative positions of the Moon, Earth, and Sun [27]. Some sources describe this cycle in terms of four primary phases: the New Moon, First Quarter, Full Moon, and Last Quarter. The New Moon occurs when the Moon is nearly aligned with the Sun relative to Earth, so that the Moon’s sunlit side faces away from Earth, making it essentially invisible to observers. The Full Moon occurs when the Moon is on the opposite side of Earth from the Sun, so that the side facing Earth is completely illuminated. The two intermediate phases in this classification (First Quarter and Last Quarter) are often called quarters, each representing a point where roughly half of the Moon’s disk is illuminated [31].

When the cycle is divided into eight phases, four additional phases are included: the Waxing Crescent, when approximately one quarter of the Moon is illuminated; the Waxing Gibbous, when approximately three quarters are illuminated; the Waning Gibbous, also with approximately three quarters illuminated but on the opposite side; and the Waning Crescent, when approximately one quarter is illuminated, on the opposite side from the Waxing Crescent. The Moon is considered waxing from New Moon to Full Moon, and waning from Full Moon to New Moon [31].

Another studied revolution is the tropical month, which lasts 27.32 days. This revolution refers to the Moon’s position in the sky and is divided into two phases: the Ascending Moon, when it appears higher in the sky relative to the horizon, and the Descending Moon, when it descends toward the horizon [32,33].

This study aimed to determine whether lunar phases are associated with calving frequency in Montbéliarde dairy cows, mostly from the Franche-Comté region of France (98.8%). The analysis was based on a large dataset spanning three years of calving records. The association between lunar phases and calving frequency in primiparous and multiparous cows, as well as in male and female calves, was also examined. Based on the observed trends, such findings may have practical implications for veterinarians and breeders, allowing optimization of management during calving periods.

## 2. Materials and Methods

### 2.1. Sample Characteristics

This retrospective study was conducted using a secondary database provided by the French Institute of Livestock (EDE) of Franche-Comté (Paris, France), under the supervision of the Ministry of Agriculture and Food Sovereignty (Paris, France). The data collected for the study comprised calvings registered over three years, from 1 March 2022 to 3 March 2025, based on 801,977 records involving 55 different cow breeds and 5434 Montbéliarde cattle farms.

From the initial dataset, which included animal number, sex, dam number, calving order, breed type of the animal, dam, and sire, only Montbéliarde breed animals were selected, totaling 393,451 records. The selection was based on breed type, with calves and parents all identified as Montbéliarde, with breed code 46. This selection aimed to ensure genetic purity and a more homogeneous and well-defined population, avoiding breed interference in statistical analyses.

Franche-Comté comprises four departments: Doubs, Jura, Haute-Saône, and Territoire de Belfort. The data analyzed represented 98.2% of calvings from Franche-Comté departments, with Doubs being the most represented, accounting for 343,263 birth records, of which 207,161 were from Montbéliarde cows, followed by Jura (*n* = 122,875), Haute-Saône (*n* = 55,014), Territoire de Belfort (*n* = 3561), and others (*n* = 4840). Of the 393,451 Montbéliarde calving dates analyzed, 126,678 corresponded to primiparous cows, and 266,772 to multiparous cows; one record with unknown calving order was excluded from the analysis. Of these calving dates, 238,066 corresponded to female calves and 155,385 to male calves.

The initial data were provided in a Microsoft Excel^®^ (version 2604) file [34] directly by EDE, Franche-Comté. During calving, breeders digitally record information about the animal, such as calving date, sex, and breed type, which are collected and stored by EDE, a structure that is part of the Chamber of Agriculture, responsible for assisting breeders in managing and monitoring their herds. EDE collects zootechnical and health data, particularly for animal identification and traceability, mainly in accordance with Article 6 of the Order of 6 August 2013 [35] regarding the identification of bovine animals.

Due to the large number of dairy farms, detailed information on herd reproductive management was not available. Therefore, possible variations in reproductive strategies among farms could not be accounted for, and this limitation should be considered when interpreting the results.

### 2.2. Characteristics of the Lunar Cycle

There are different ways to describe the lunar cycle. Each date in the Gregorian calendar was assigned a corresponding day or phase of the synodic lunar cycle, using synodic lunar time obtained directly from the National Aeronautics and Space Administration (NASA) website at 10:00 UTC, which corresponds to 12:00 (UTC+2) in France [36].

To ensure the same number of synodic lunar days in each complete cycle (*n* = 36), the study was conducted from 3 March 2022 to 29 January 2025, covering 383,926 calvings. The data were initially distributed by day of the lunar cycle, and the synodic lunar days were defined using equal intervals of 1.02 days (29.53 ÷ 29 = 1.02), ranging from 1 to 29 (day 29 includes days above 28.56), ensuring a homogeneous distribution across the lunar cycle.

**Figure 1:**
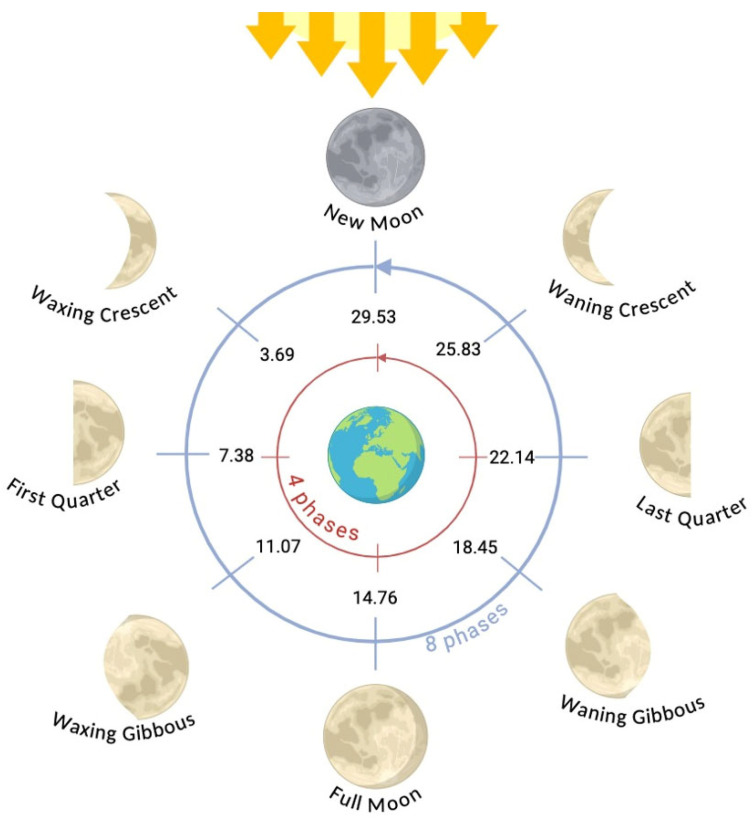
Division of the different phases of the lunar cycle in days (original source by the author, created with Biorender System).

The data were then divided into four and eight phases of the lunar cycle, homogeneously separating the phases along the lunar calendar, based on the average lunar cycle duration of 29.53 days (Figure 1), and also accounting for the waxing and waning phases. The four phases were categorized as follows: New Moon (days 0 to 3.69 and above 25.83, *n* = 96,611); First Quarter (days 3.69 to 11.07, *n* = 95,626); Full Moon (days 11.07 to 18.45, *n* = 95,693); Last Quarter (days 18.45 to 25.83, *n* = 95,996). The eight phases were categorized as follows: New Moon to Waxing Crescent (days 0 to 3.69, *n* = 49,958); Waxing Crescent to First Quarter (days 3.69 to 7.38, *n* = 46,653); First Quarter to Waxing Gibbous (days 7.38 to 11.07, *n* = 48,126); Waxing Gibbous to Full Moon (days 11.07 to 14.76, *n* = 47,500); Full Moon to Waning Gibbous (days 14.76 to 18.45, *n* = 46,918); Waning Gibbous to Last Quarter (days 18.45 to 22.14, *n* = 49,061); Last Quarter to Waning Crescent (days 22.14 to 25.83, *n* = 47,173), and Waning Crescent to New Moon (days above 25.83, *n* = 48,537).

Subsequently, the impact of the ascending and descending phases of the Moon was examined. These phases represent the lunar declination cycle, also known as the tropical month, and are not part of the synodic lunar cycle [33]. A cross-reference table was used to assign each Gregorian calendar date the corresponding information on whether the Moon was ascending or descending, according to a lunar calendar [37].

### 2.3. Statistical Analysis

Data summaries were performed using Microsoft Power BI Desktop^®^ (version 2.144.679.0) [38], based on information extracted from the Microsoft Excel^®^ (version 2604) [34] database and formulas applied for calculations associated with lunar cycles.

Statistical analyses were subsequently performed in R (version 4.4.3) [39], using the Generalized Linear Model (GLM) [40], with Lunar variables (synodic day, four-phase lunar cycle, eight-phase lunar cycle, waxing/waning phases and ascending/descending phases), farm region, sex, parity (primiparous/multiparous), year and season, all as fixed effects. A total of 9228 combinations of factors were analyzed, resulting from the aggregation of calvings by farm region, sex, parity, year, season and lunar variable.

For each lunar variable, a GLM with a log link function was specified with the following structure: Number of Calvings ~ Lunar Variable + Region + Year + Season + Sex + Parity.

The models were initially fitted with a Poisson distribution with log link and subsequently, due to overdispersion detection (Pearson *χ*^2^/*df* > 1.4), with a Negative Binomial distribution with log link. Additionally, interactions between each lunar variable and the moderating covariates were assessed using likelihood ratio tests (LRT), comparing models with and without interaction terms.

The choice of statistical contrasts was defined according to the nature of the variables. Deviation contrasts were applied to all variables, comparing each category against the overall mean rather than a reference category, thereby eliminating reference selection bias and enabling assessment of deviations from expected average behavior. For models including interactions, effects were estimated within each level using marginal contrasts. As raw coefficients from models including interactions are not directly interpretable, inference was based on adjusted marginal means (emmeans). The adjusted means were computed on the response scale and subsequently transformed into Incidence Rate Ratios (IRR) by contrasting each lunar level with the overall marginal mean of the factor on the log-link scale. Corresponding confidence intervals (95% CI) and *p*-values were obtained from Wald tests performed on the log scale. Type III Wald tests and Likelihood Ratio Tests (LRT) were used to assess the global significance of each term. A significance level of *p* < 0.05 was considered.

## 3. Results

The analysis of the GLM revealed significant contributions from all main fixed effects (region, sex, parity, year and season), which showed statistical significance across all lunar models (*p* < 0.001). Regarding the lunar variables, significant associations were detected, particularly for the synodic lunar day and the multi-phase groupings (four-phase and eight-phase lunar cycles), although the magnitude of these effects differed according to the level of lunar aggregation. In contrast, binary lunar classifications (waxing/waning phases and ascending/descending phases) did not show a statistically significant influence on calving frequency. Interaction analyses showed that the association between the lunar cycle and calving frequency was significantly modulated by season for the synodic lunar day, four-phase and eight-phase lunar cycles (*p* < 0.001), and to a lesser extent for the waxing/waning cycle (*p* < 0.05). However, no interactions were detected between lunar variables and either sex or parity (*p* > 0.05), indicating that while these covariates significantly influence the overall calving frequency, they do not modulate the association between lunar cycles and calving frequency.

### 3.1. Monthly Distribution of Calving in Montbéliarde Cows

A descriptive analysis showed that the distribution of calving dates was not uniform throughout the months of the year, likely reflecting seasonal influences.

**Figure 2:**
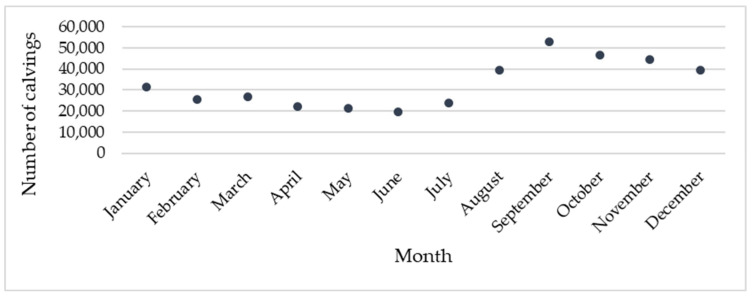
Distribution of calvings by month from 1 March 2022 to 28 February 2025, representing three complete calendar years. Values represent cumulative calving counts across this period. Note: this period differs slightly from the study period used in the statistical analysis (3 March 2022 to 29 January 2025), which was defined to ensure an equal number of complete synodic lunar cycles.

Data from 1 March 2022 to 28 February 2025 showed that the number of calvings was highest in September, with 52,963 parturitions, and lowest in June, with 19,489 parturitions, as illustrated in Figure 2.

The distribution showed a higher concentration of calvings beginning in late summer, remaining elevated during autumn, and declining in winter. In addition to the natural fluctuation of fertility rates in cows, reproductive management practices are implemented within herds to concentrate calvings during periods that favor calf rearing and enhance milk production at specific times of the year.

**Figure 3:**
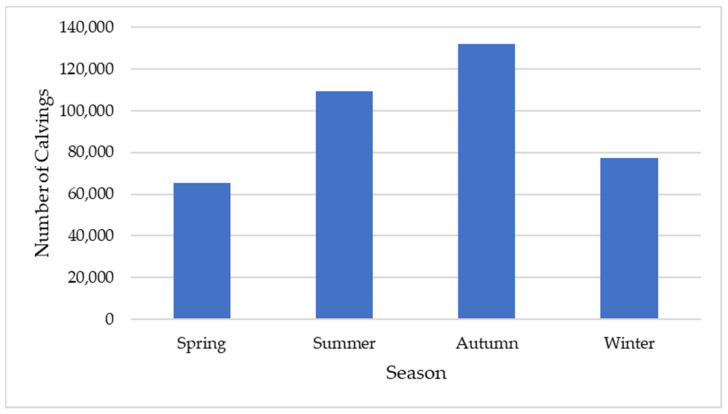
Distribution of calvings by season from 3 March 2022 to 29 January 2025. Values represent cumulative calving counts across the full study period (*n* = 383,926).

The seasonal distribution for the studied dataset, from 3 March 2022 to 29 January 2025, is presented in Figure 3.

### 3.2. Analysis by Synodic Lunar Days

**Figure 4:**
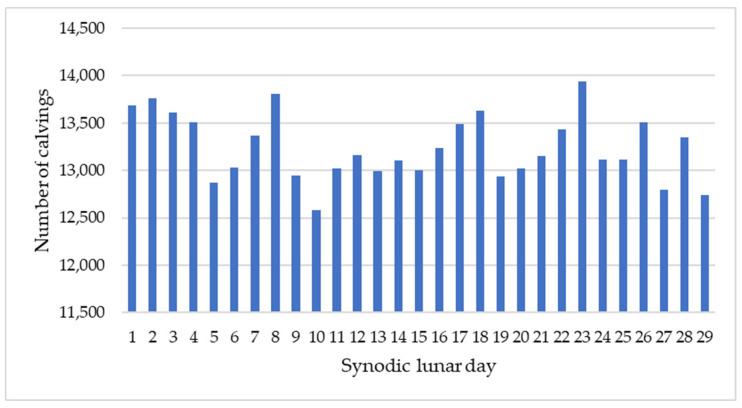
Distribution of calvings across the 29-day synodic lunar cycle from 3 March 2022 to 29 January 2025. Values represent cumulative calving counts across the full study period (*n* = 383,926).

The data indicate that calvings are not uniformly distributed across synodic lunar days, as shown in Figure 4.

**Figure 5:**
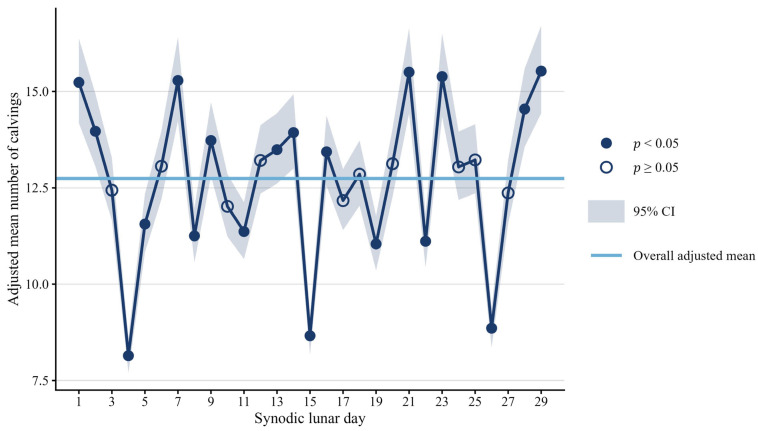
Model-adjusted mean number of calvings throughout the 29-day synodic lunar cycle. Adjusted means represent the expected number of calvings per combination of covariate levels, estimated by a negative binomial GLM controlling for region, year, season, sex and parity. These values are not directly comparable to raw calving counts, which are expressed in the order of tens of thousands.

Analysis of the model-adjusted means estimated while controlling for region, year, season, sex, and parity revealed marked oscillating patterns throughout the synodic lunar cycle, as shown in Figure 5.

Several peak periods with significantly elevated calving frequency (*p* < 0.001) were observed throughout the synodic lunar cycle, with day 29—near the New Moon—showing the highest adjusted mean, exceeding the overall adjusted mean by 23.4%, followed by day 21 (+23.2%), near the Last Quarter. Days 1 and 28, near the New Moon, also showed significantly elevated calving frequency (*p* < 0.001), with values exceeding the overall mean by more than 15%. In contrast, day 4, near the Waxing Crescent, presented the lowest value (35.2% below the overall mean), followed by day 15 (−31.1%), near the Full Moon (both *p* < 0.001). Days 8, 19, 22, and 26 also presented a significantly reduced calving frequency (*p* < 0.001), with values ranging from 10.6% to 29.6% below the overall mean.

#### 3.2.1. Analysis by Synodic Lunar Days Versus Sex and Parity

Differences between primiparous and multiparous cows, as well as comparisons between male and female calves, are presented in Table 1. Both subgroups followed the same overall pattern observed for the total sample.

Synodic days 1 and 29, both near the New Moon, and days 21 and 23, near the Last Quarter, consistently showed the highest adjusted mean across all subgroups, exceeding the overall mean by more than 19% (*p* < 0.01). Days 4 (near the Waxing Crescent) and 15 (near the Full Moon) presented the greatest reductions, with values at least 34% below the overall mean (*p* < 0.001).

#### 3.2.2. Analysis by Synodic Lunar Days Versus Season

**Figure 6:**
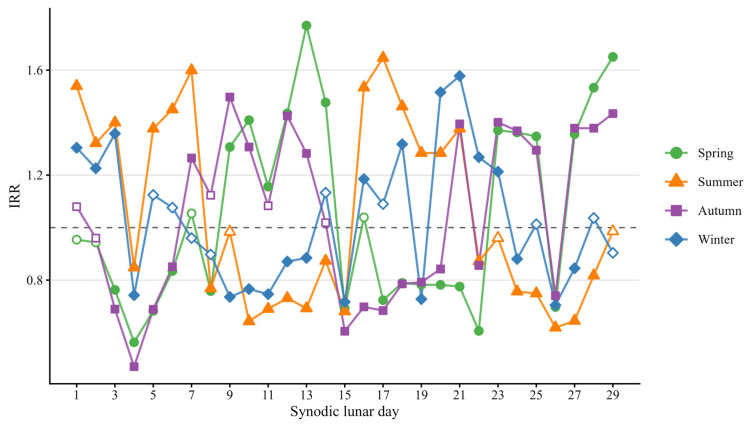
Season-stratified IRR across the 29-day synodic lunar cycle. Filled symbols indicate values significantly different from the overall mean (*p* < 0.05); and open symbols indicate non-significant differences (*p* ≥ 0.05). The dashed line represents IRR = 1.

The season-stratified IRR profiles across the synodic lunar cycle are presented in Figure 6, illustrating the variation in calving frequency by lunar day each season.

The highest magnitude of lunar effect was observed in Spring (ranging from −43.7% to +77%), with several synodic days showing significantly elevated calving frequency. The most pronounced peak occurred on synodic day 13 (+77%), near the Full Moon, followed by day 29 (+65%), near the New Moon. Days 10, 12, 14, and 28 also presented significantly elevated adjusted mean (≥+40%). The largest negative deviations were observed on day 4 (−43.7%), near the Waxing Crescent, and day 22 (−39.4%), near the Last Quarter.

Summer presented a well-defined pattern with strong positive deviations (≥+30%), on days 1–3 and 5–7, between the New Moon and First Quarter, and on days 16–21, between the Full Moon and Last Quarter. Negative deviations (<−12%) were observed on days 8–15, between the First Quarter and Full Moon, and on days 22–29, between the Last Quarter and New Moon.

Autumn presented the most asymmetrical pattern, with scattered increases and more concentrated reductions. The highest positive deviation occurred on day 9 (+49.7%), after the First Quarter. Days 27–29, near the New Moon, also present elevated calving frequency (≥+37%). The most notable negative deviation occurred during this season, on day 4 (−53%), near the Waxing Crescent.

Overall, Winter presented fewer significant days but showed elevated calving frequency on days 1–3 (≥+22%), between the New Moon and Waxing Crescent, and on days 20–23 (≥+21%), between the Waning Gibbous and Waning Crescent, with notable peaks on days 20 and 21 (>+51%). Days 15 and 26 showed negative deviations (<−28%), near the Full Moon and Waning Crescent respectively.

### 3.3. Analysis by Lunar Phases

#### 3.3.1. Distribution in Eight Phases of the Synodic Revolution

**Figure 7:**
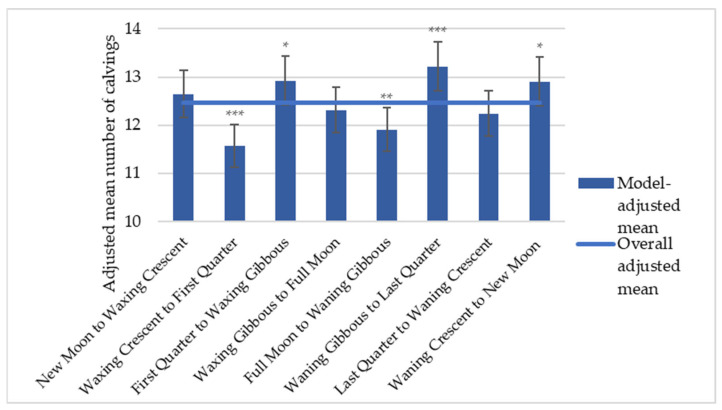
Model-adjusted mean number of calvings across the eight-phase lunar cycle. Error bars represent 95% CI. * Statistically significant for a *p*-value < 0.05, ** Statistically significant for a *p*-value < 0.01, *** Statistically significant for a *p*-value < 0.001. Adjusted means represent the expected number of calvings per combination of covariate levels, estimated by a negative binomial GLM controlling for region, year, season, sex and parity. These values are not directly comparable to raw calving counts, which are expressed in the order of tens of thousands.

Analysis of the eight-phase lunar cycle further supported the association between the lunar phase and calving frequency (Figure 7), consistent with the pattern observed for synodic days.

The model analysis demonstrated significantly elevated calving frequency during the Waning Gibbous to Last Quarter phase (+6.2%, *p* < 0.001), the First Quarter to Waxing Gibbous phase (+3.7%, *p* < 0.05), and the Waning Crescent to New Moon phase (+3.6%, *p* < 0.05), all exceeding the overall adjusted mean. Conversely, significantly reduced calving frequency was observed during the Waxing Crescent to First Quarter phase (−7.11%, *p* < 0.001), and to the Full Moon to Waning Gibbous phase (−4.4%, *p* < 0.01).

**Figure 8:**
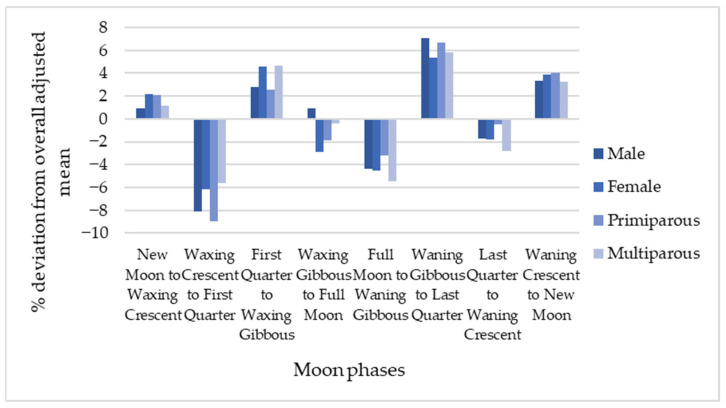
Percentage change in calving frequency for eight-phase lunar cycle in primiparous and multiparous cows and on the calving of males and females.

Similar patterns were observed across all subgroups, male and female calves, and primiparous and multiparous cows, as illustrated in Figure 8, although some differences in statistical significance were noted. For phases with elevated calving frequency, the association with the Waning Gibbous to Last Quarter phase was statistically significant across all subgroups (*p* < 0.05), with the exception of female calves. In contrast, no statistically significant associations were detected for the First Quarter to Waxing Gibbous and Waning Crescent to New Moon phases. Regarding phases with reduced calving frequency, the Waxing Crescent to First Quarter phase was significant across all subgroups (*p* < 0.05), whereas the Full Moon to Waning Gibbous phase reached statistical significance only in multiparous cows (*p* < 0.05).

In the eight-phase lunar analysis, specific phases associated with significant increases or decreases in calving frequency were identified across seasons, as presented in Table 2.

Across the eight-phase lunar cycle, clear seasonal differences were observed in the pattern of calving frequency. Spring and Autumn displayed the strongest contrasts, with marked increases during the First Quarter to Waxing Gibbous phase and the Waning Crescent to New Moon phase (>30%, *p* < 0.001), and consistent reductions during the New Moon to Waxing Crescent (≤−14%, *p* < 0.001), the Waxing Crescent to First Quarter (≤−18%, *p* < 0.001) and the Full Moon to Waning Gibbous phase (≤−15%, *p* < 0.01).

Summer presented the most systematic pattern, with elevated calving frequency during the early waxing and waning phases (*p* < 0.001)—from the New Moon to the First Quarter (≥16%), and from the Full Moon to the Last Quarter (≥31%). Presented pronounced reductions during the late waxing and waning phases (*p* < 0.001)—from the First Quarter to the Full Moon (≤−20%) and from the Last Quarter to the New Moon (≤−22%).

Winter showed similar but more moderate fluctuations, with increased calving frequency during the early waxing and waning phases—from the New Moon to Waxing Crescent (14.8%, *p* < 0.001) and from the Waning Gibbous to Last Quarter (24.9%, *p* < 0.001)—and a reduction during the Waning Crescent to New Moon phase (−11.3%, *p* < 0.001). Overall, these results indicate that the association between the eight-phase lunar cycle and calving frequency follows a seasonally modulated pattern.

#### 3.3.2. Distribution in Four Phases of the Synodic Revolution

**Figure 9:**
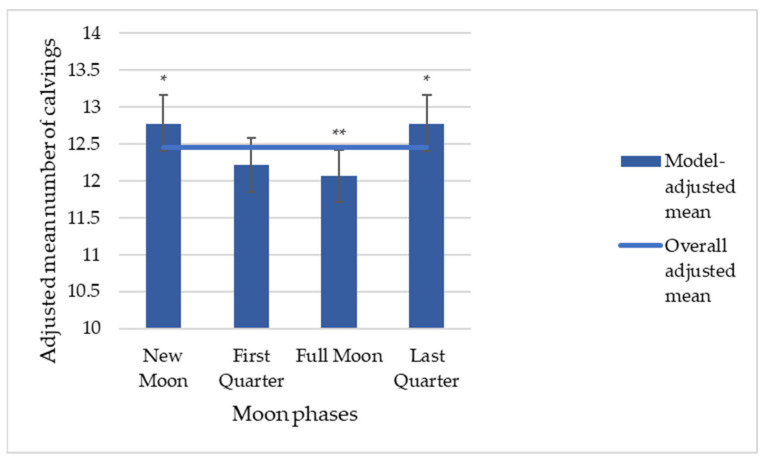
Model-adjusted mean number of calvings across the four-phase lunar cycle. Error bars represent 95% CI. * Statistically significant for a *p*-value < 0.05, ** Statistically significant for a *p*-value < 0.01. Adjusted means represent the expected number of calvings per combination of covariate levels, estimated by a negative binomial GLM controlling for region, year, season, sex and parity. These values are not directly comparable to raw calving counts, which are expressed in the order of tens of thousands.

When the synodic lunar cycle was divided into four phases, a similar but less detailed pattern was observed. The adjusted mean revealed significantly elevated calving frequency during the New Moon and Last Quarter phases (+2.6%, *p* < 0.05) and a significant reduction during the Full Moon phase (−3%, *p* < 0.01), as illustrated in Figure 9. No statistically significant difference was detected for the First Quarter phase (*p* > 0.05).

**Figure 10:**
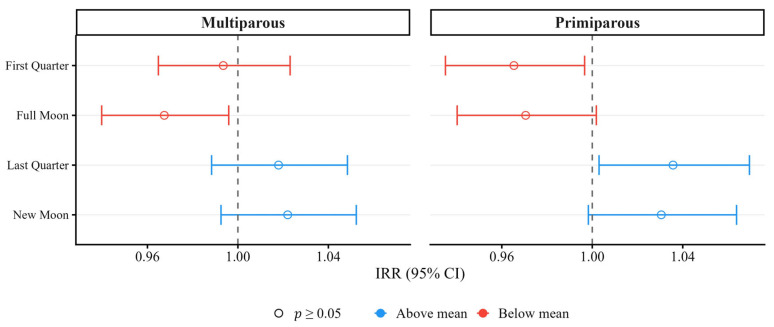
IRR with 95% CI for the four-phase lunar cycle, stratified by parity.

A similar association trend was observed in both primiparous and multiparous cows, as illustrated in Figure 10, although no statistically significant differences were observed (*p* > 0.05).

**Figure 11:**
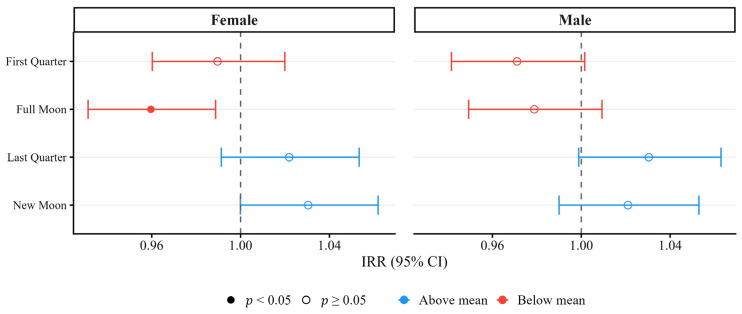
IRR with 95% CI for the four-phase lunar cycle, stratified by calf sex.

A similar analysis was performed for male and female calves (Figure 11), revealing the same overall association trend. However, statistical significance was reached only for female calves during the Full Moon phase (−4%, *p* < 0.05).

Further stratification by season revealed distinct patterns in the association between the four-phase lunar cycle and calving frequency (Table 3). In Spring, significantly elevated calving frequency was observed during the New Moon (+5.7%, *p* < 0.05) and Full Moon phases (+5.3%, *p* < 0.05), while a significant reduction was detected during the Last Quarter phase (−6.7%, *p* < 0.01). In Autumn, calving frequency was significantly elevated during the First Quarter (+7%, *p* < 0.01) and Last Quarter phases (+5.3%, *p* < 0.05) and significantly reduced during the Full Moon phase (−12.2%, *p* < 0.001). In Winter, a significant increase was observed during the Last Quarter phase (+14.9%, *p* < 0.001), while a significant reduction was detected during the First Quarter phase (−9.7%, *p* < 0.001). In Summer, no statistically significant association between four-phase lunar cycle and calving frequency was detected.

Across all seasons, a consistent trend toward increased calving frequency was observed during the New Moon phase, reaching statistical significance only in Spring (+5.7%, *p* < 0.05).

Overall, all models demonstrated significant association between the four-phase lunar cycle and calving frequency, with a tendency for increased frequency during the New Moon phase and reduced frequency during the First Quarter or Full Moon phases.

#### 3.3.3. Synodic Waxing and Waning Phases

Although no significant difference in calving frequency was detected between the waxing and waning phases at the global level, significant seasonal variability was observed (*p* < 0.05). A clear crossover interaction was identified: during Winter, the waning phase was associated with increased calving frequency (+4.9%), while the waxing phase was associated with a decrease (−4.6%). This pattern was reversed or neutralized during other seasons, such as Spring. This trend inversion explains the absence of a significant main effect in the global analysis, as the opposing seasonal effects cancel each other out when aggregated across seasons.

#### 3.3.4. Analysis by the Tropical Month

Analysis of the tropical month revealed no significant association between the ascending or descending lunar phase and calving frequency (*p* > 0.05). Unlike the synodic cycle, the tropical month did not appear to modulate parturition timing in the studied population.

## 4. Discussion

The results obtained in this study, to some extent, corroborate findings reported in the literature, especially in studies conducted with dairy cows [1] and dual-purpose cows [3,4,5], in which statistical associations between lunar variables and the temporal distribution of calvings were also identified. However, these findings are based on an observational study and should be interpreted with caution due to its nature.

A study on the bovine population of Switzerland, with more than two million calvings over three years, showed that the frequency of calvings per day was statistically higher between days 13 and 15 of the lunar cycle, and lower between days 9 and 12 [4]. In the present study, the synodic days with the highest calving frequency were days 21 and 29, while days 4 and 15 presented the lowest frequency.

In a study conducted with 5869 calvings from crossbred cows in Venezuela, statistical analysis showed a higher concentration of calvings around the New Moon and Full Moon [3]. Another study, also carried out in Venezuela, with 121,276 parturitions from crossbred cows across 36 farms, similarly identified a statistically significant peak concentration around these two phases [5].

A study carried out solely with Holstein dairy cows showed that, in 428 calvings on a farm in Hokkaido over three years, there was a statistically significant increase in calvings from the New Moon to the Full Moon, followed by a decrease toward the Waning Crescent [1]. However, even though the Full Moon phase appears to be the phase with the highest concentration of calvings in the previously cited studies, this was not the case in the present study, which analyzed 383,926 calvings in the Franche-Comté region, France. In fact, the number of calvings in Montbéliarde dairy cows was highest during the New Moon phase, with 98,495 calvings, when the cycle was divided into four phases. Given the large sample size, the study has high statistical power to detect even small differences. Therefore, statistically significant results may be associated with modest effect sizes, which should be interpreted in context, particularly considering the consistent patterns observed across biological subgroups, including primiparous and multiparous cows, as well as male and female calves. These subgroups showed similar trends in the distribution of calvings in relation to synodic days, reinforcing the robustness of the observed associations despite their modest magnitude.

A similar association trend was also observed, with calving frequency increasing during the New Moon in Spring, while in Winter the Last Quarter showed the highest distribution. Season-stratified IRR patterns across the synodic lunar cycle revealed that the magnitude and expression of lunar-associated variations differed markedly between seasons. Spring showed the greatest variability, with several significant increases and decreases in calving frequency across synodic days, whereas Winter exhibited smaller fluctuations and fewer significant effects, with Summer and Autumn displaying intermediate patterns. Notably, the timing and direction of these lunar-related deviations were not consistent across seasons, indicating that the influence attributed to the lunar cycle is not uniform but varies according to seasonal conditions.

These findings are consistent with previous studies reporting that lunar effects on calving are generally modest and inconsistent, with variations in calving frequency observed across the lunar cycle but lacking stable patterns across populations [1,5]. Moreover, environmental factors, such as temperature and other meteorological conditions, have been shown to exert a stronger and more consistent influence on the onset of parturition than lunar phases [41]. Although recent studies suggest potential associations between lunar phases and reproductive parameters, including calving frequency and gestation length, these effects remain context dependent and mechanistically unclear [3,5]. In this context, the present results support the interpretation that lunar-associated patterns are likely modulated by seasonal conditions and should therefore be interpreted within a broader environmental framework rather than as an independent effect.

Regarding the discrepancy in birth rates between males (39.5%) and females (60.5%), it is important to note that there is no available information in this study concerning the use of reproductive techniques such as sexed semen, which could explain such a difference. This represents a limitation of the study.

In beef cattle, however, it was not possible to corroborate the results obtained in this study. Indeed, a study conducted in Japan with 41,116 calvings showed no association between lunar phases and the number of calvings [6].

To date, most studies on the association of the Moon with parturitions have been conducted in humans. The results of some studies indicate the absence of an association between the lunar cycle and parturitions according to some authors [42,43,44,45,46,47]. However, other studies report a relationship with the Moon, but without consensus on the phase involved. In an Austrian study, an increase in parturitions was noted between the Waxing Crescent and the First Quarter [48]. Another Iraqi study found an increase in parturitions during the Full Moon and the Waning Gibbous phase [49]. In a French study with approximately 30 million parturitions, a significant, albeit weak, Full Moon effect was identified [50]. In the present study, non-significant findings were observed for certain lunar variables, such as waxing/waning and ascending/descending phases. These results are not unexpected and align with the broader literature. Notably, the classification of lunar phases into ascending and descending cycles has been scarcely investigated in the context of cattle reproduction, with most studies focusing on the synodic cycle or lunar luminosity. Therefore, the absence of significant associations for these variables likely reflects both the limited biological evidence supporting these subdivisions and the inherent variability of lunar-related patterns. These results should thus be interpreted as part of the overall heterogeneity of the phenomenon.

The nighttime calving rate varies from 24% to 54% [51]. This remains a study hypothesis that may be associated with the observed increase in calvings during the New Moon phase, when nocturnal lunar luminosity is reduced. The present study suggests that parturitions occur more frequently during a phase in which nocturnal atmospheric illumination from the Moon is absent. However, it is important to emphasize that the data analyzed correspond to dates of birth with no time recorded, representing a study limitation.

The behavioral phenomenon of pregnant animals responding to decreased luminosity seems to be a plausible hypothesis. During bovine calving, the cow’s behavior changes and she isolates herself [13], and low lighting conditions may represent an advantage during calving for self-protection. Indeed, during parturition in wild animals, they attempt to hide to avoid predators. In ruminants, they isolate themselves from the rest of the herd to be less visible and hide in secluded topographical areas [52]. Due to the docile temperament of the Montbéliarde cow [18], concealment behavior during parturition does not appear to be typical; however this was not measured in this study.

Another hypothesis may be related to the secretion of melatonin, which varies as a function of light and the lunar cycle [1,3,53]. Studies on the Moon’s association with cattle suggest that nighttime melatonin secretion is associated with lunar phases, with a peak around the New Moon [1,4]. This hormone, present in the ovaries and myometrium of cows, may play a role in triggering calving [5]. In humans, an increase in melatonin levels was observed at the end of gestation, followed by a marked decrease at the time of birth [1]. In addition, melatonin production is higher at the beginning of the estrous cycle, a period centered around the New Moon [1,3,5,53]. Seasonal stratification of synodic days revealed distinct temporal patterns, indicating that the magnitude and direction of the lunar effect are not uniform throughout the year. A consistent trend toward increased calving frequency was observed during the New Moon phase across all seasons, reaching statistical significance only in Spring (*p* < 0.05). A study conducted in Venezuela analyzed the seasonality of calvings; however, no significant association was found [3]. Additional studies are required to better understand the association between the Moon and seasonal effects on calving.

In summary, the association of gravitational forces during the day and the absence of lunar luminosity at night represents another hypothesis that could potentially explain the significant increase in the number of calvings associated with the New Moon. Further studies are needed to better understand the associations found with the different lunar revolutions.

Knowledge of this variation in the number of bovine calvings throughout the different phases of the lunar cycle could help veterinarians and breeders in this region, who predict births based on lunar phases, to optimize herd management during the calving period and improve colostrum administration, thereby reducing failures in passive transfer of immunity as well as the associated economic losses [23]. For adequate absorption of maternal immunoglobulins present in colostrum, administration must be early, ideally within 0 to 2 h of birth and mandatorily before 24 h, in sufficient quantity (3 to 4 L at first administration) and over an adequate duration of at least 15 min [23,24,25].

It is important to emphasize that the absence of additional parameters in this study, such as weather conditions, time of calving, dates of conception, use of artificial insemination, farm altitude, and stress factors, represents a limitation, preventing the establishment of a more precise reproductive framework. Additionally, alternative model specifications were explored during the analyses. However, some configurations resulted in convergence failures, likely attributable to the small number of observations within certain subgroups and the final model was therefore selected based on both theoretical considerations and computational feasibility. These variables and constraints could be addressed in future studies.

## 5. Conclusions

This baseline study carried out with Montbéliarde cows in the Franche-Comté region demonstrated an association between the distribution of calvings and the four phases of the lunar cycle, with higher calving frequency during the New Moon phase.

The significance of fixed covariates (region, season, year, calf sex and parity) was consistent across all models confirming biological and herd-level variability. No significant interaction was detected between lunar variables and calf sex, with the exception of female calves during the Full Moon, or parity; however, a significant interaction was detected for season. This indicates that the association between the synodic lunar cycle and calving frequency is not uniform throughout the year, showing distinct seasonal modulations in both the magnitude and direction of effects. The overall heterogeneity of the phenomenon, particularly across seasons, should therefore be taken into consideration.

These findings may offer exploratory insights for breeders in this region regarding potential variations in calving distribution, which could be considered when planning colostrum management, particularly in larger herds. However, these observations should not be interpreted as direct management recommendations. Further prospective studies incorporating additional variables, such as the use of reproductive biotechnologies, hormonal profiles, and environmental factors, are needed to better clarify these associations.

## Figures and Tables

**Table 1:** Association between synodic lunar days and calvings for primiparous and multiparous cows and for male and female calves.

	PP (Day)	IRR	IRR 95% CI	PE	NP (Day)	IRR	IRR 95% CI	PE
Primiparous	1	1.19 **	1.08–1.32	19.3%	4 8 11 15	0.65 ***	0.60–0.71	−34.8%
	2	1.13 *	1.02–1.24	12.6%		0.89 *	0.82–0.98	−10.7%
	7	1.18 **	1.07–1.31	18.2%		0.88 *	0.81–0.97	−11.6%
	21	1.24 ***	1.13–1.38	24.4%		0.69 ***	0.63–0.75	−31.4%
	23	1.24 ***	1.12–1.37	24.0%	19	0.88 *	0.81–0.97	−11.8%
	28	1.18 **	1.07–1.30	17.9%	22	0.89 *	0.81–0.97	−11.2%
	29	1.26 ***	1.14–1.40	26.2%	26	0.70 ***	0.65–0.76	−29.5%
Multiparous	1	1.23 ***	1.12–1.35	22.7%	4	0.64 ***	0.60–0.69	−35.6%
	7	1.24 ***	1.13–1.36	24.3%	8	0.90 *	0.83–0.97	−10.4%
	21	1.22 ***	1.11–1.34	22.2%	15	0.69 ***	0.64–0.74	−30.9%
	23	1.21 ***	1.10–1.33	20.9%	19	0.87 **	0.80–0.95	−12.6%
	28	1.14 *	1.04–1.25	13.6%	22	0.88 **	0.81–0.95	−12.1%
	29	1.21 ***	1.10–1.33	21.2%	26	0.70 ***	0.65–0.76	−29.6%
Males	1	1.20 **	1.08–1.32	19.6%	4	0.64 ***	0.60–0.69	−35.7%
	7	1.23 ***	1.11–1.35	22.7%	5	0.89 *	0.81–0.97	−11.2%
	14	1.15 **	1.05–1.27	15.1%	8	0.88 **	0.81–0.96	−12.2%
	21	1.25 ***	1.14–1.38	25.4%	11	0.89 *	0.81–0.97	−11.3%
	23	1.26 ***	1.14–1.39	25.8%	15	0.68 ***	0.63–0.74	−31.5%
	28	1.16 **	1.06–1.28	16.3%	19	0.88 *	0.81–0.97	−11.6%
	29	1.21 **	1.09–1.33	20.6%	22	0.87 **	0.80–0.95	−12.6%
					26	0.71 ***	0.66–0.77	−28.8%
Females	1	1.23 ***	1.11–1.35	22.5%	4	0.65 ***	0.61–0.70	−34.8%
	2	1.12 *	1.02–1.22	11.7%	15	0.69 ***	0.64–0.75	−30.8%
	7	1.20 **	1.10–1.32	20.4%	19	0.87 **	0.80–0.95	−12.8%
	21	1.21 ***	1.10–1.33	21.1%	22	0.89 *	0.82–0.97	−10.8%
	23	1.19 **	1.08–1.31	19.2%	26	0.70 ***	0.65–0.75	−30.2%
	28	1.15 ***	1.05–1.26	14.9%				
	29	1.26 **	1.14–1.39	26.1%				

* Statistically significant for a *p*-value < 0.05, ** Statistically significant for a *p*-value < 0.01, *** Statistically significant for a *p*-value < 0.001. Type III tests overall *p*-values: Synodic Day *p* = 1.5 × 10^−153^; Department *p* < 2 × 10^−16^; Season *p* < 2 × 10^−16^; Year = 7.4 × 10^−77^; Sex *p* = 1.1 × 10^−170^; Parity *p* < 2 × 10^−16^. Legend: PP—Positive Probability; IRR—Incidence Rate Ratio; PE—Percentual Effect; CI—Confidence Intervals; NP—Negative Probability. All days of the synodic lunar cycle were analyzed; however, only those showing statistically significant differences from the overall adjusted mean are presented in the table.

**Table 2:** Association between eight-phase lunar synodic cycle and calvings by season.

	PP (Phase)	IRR	IRR 95% CI	PE	NP (Phase)	IRR	IRR 95% CI	PE
Spring	First Quarter to Waxing Gibbous	1.30 ***	1.21–1.40	30.1%	New Moon to Waxing Crescent	0.85 ***	0.80–0.90	14.9%
	Waxing Gibbous to Full Moon	1.29 ***	1.21–1.38	29.3%	Waxing Crescent to First Quarter	0.73 ***	0.68–0.77	26.9%
	Last Quarter to Waning Crescent	1.09 *	1.02–1.16	8.9%	Full Moon to Waning Gibbous	0.84 ***	0.79–0.89	16%
	Waning Crescent to New Moon	1.33 ***	1.24–1.42	32.7%	Waning Gibbous to Last Quarter	0.79 ***	0.74–0.83	21.4%
Summer	New Moon to Waxing Crescent	1.39 ***	1.30–1.49	39.0%	First Quarter to Waxing Gibbous	0.79 ***	0.75–0.84	20.8%
	Waxing Crescent to First Quarter	1.16 ***	1.09–1.24	16.2%	Waxing Gibbous to Full Moon	0.76 ***	0.72–0.80	23.9%
	Full Moon to Waning Gibbous	1.32 ***	1.24–1.41	31.8%	Last Quarter to Waning Crescent	0.76 ***	0.72–0.80	23.9%
	Waning Gibbous to Last Quarter	1.32 ***	1.24–1.41	32.1%	Waning Crescent to New Moon	0.77 ***	0.73–0.82	22.5%
Autumn	First Quarter to Waxing Gibbous	1.40 ***	1.31–1.50	40.3%	New Moon to Waxing Crescent	0.79 ***	0.75–0.84	20.7%
	Waxing Gibbous to Full Moon	1.12 ***	1.05–1.19	11.8%	Waxing Crescent to First Quarter	0.81 ***	0.76–0.86	19%
	Last Quarter to Waning Crescent	1.10 **	1.03–1.17	9.5%	Full Moon to Waning Gibbous	0.69 ***	0.66–0.73	30.8%
	Waning Crescent to New Moon	1.33 ***	1.24–1.42	32.5%				
Winter	New Moon to Waxing Crescent	1.15 ***	1.08–1.22	14.8%	First Quarter to Waxing Gibbous	0.81 ***	0.76–0.85	19.4%
	Full Moon to Waning Gibbous	1.09 *	1.02–1.16	8.8%	Waxing Gibbous to Full Moon	0.87 ***	0.82–0.93	12.9%
	Waning Gibbous to Last Quarter	1.25 ***	1.17–1.34	24.9%	Waning Crescent to New Moon	0.89 ***	0.84–0.94	11.3%

* Statistically significant for a *p*-value < 0.05, ** Statistically significant for a *p*-value < 0.01, *** Statistically significant for a *p*-value < 0.001. Type III tests overall *p*-values: Eight-phases *p* = 1.6 × 10^−7^; Department *p* < 2 × 10^−16^; Season *p* ≤ 2 × 10^−16^; Year *p* 7.9 × 10^−72^; Sex *p* = 2.6 × 10^−171^; Parity *p* < 2 × 10^−16^. Legend: PP—Positive Probability; IRR—Incidence Rate Ratio; PE—Percentual Effect; CI—Confidence Intervals; NP—Negative Probability.

**Table 3:** Association between four phases of the synodic cycle and calvings by season.

	PP (Phase)	IRR	IRR 95% CI	PE	NP (Phase)	IRR	IRR 95% CI	PE
Spring	New Moon	1.06 *	1.01–1.11	5.7%	First Quarter	0.96	0.92–1.01	3.7%
	Full Moon	1.05 *	1.01–1.10	5.3%	Last Quarter	0.93 **	0.89–0.97	6.7%
Summer	New Moon	1.02	0.98–1.07	2.5%	First Quarter	0.98	0.94–1.03	1.7%
	Full Moon	1.003	0.96–1.05	0.3%	Last Quarter	0.99	0.95–1.03	1%
Autumn	New Moon	1.01	0.97–1.05	1.1%	Full Moon	0.88 ***	0.84–0.91	12.2%
	First Quarter	1.07 **	1.03–1.12	7%				
	Last Quarter	1.05 *	1.01–1.10	5.3%				
Winter	New Moon	1.01	0.97–1.05	0.9%	First Quarter	0.90 ***	0.86–0.94	9.7%
	Last Quarter	1.15 ***	1.10–1.20	14.9%	Full Moon	0.96	0.92–0.999	4.4%

* Statistically significant for a *p*-value < 0.05, ** Statistically significant for a *p*-value < 0.01, *** Statistically significant for a *p*-value < 0.001. Type III tests overall *p*-values: Four-phases *p* = 8.2 × 10^−4^; Department *p* < 2 × 10^−16^; Season *p* < 2 × 10^−16^; Year = 1.6 × 10^−71^; Sex *p* = 1.1 × 10^−170^; Parity *p* < 2 × 10^−16^. Legend: PP—Positive Probability; IRR—Incidence Rate Ratio; PE—Percentual Effect; CI—Confidence Intervals; NP—Negative Probability.

## Data Availability

The data presented in this study are available on request from the corresponding author due to official confidentiality restrictions.

## Abbreviations

The following abbreviations are used in this manuscript:

EDE	Institute of Livestock
CI	Confidence Intervals
IRR	Incidence Rate Ratio
NASA	National Aeronautics and Space Administration
NP	Negative Probability
PE	Percentual Effect
PP	Positive Probability
UTC	Coordinated Universal Time
%	Percentage
